# Feasibility and reliability of measured glomerular filtration rate with [I125]-iothalamate among young adults with mild-to-moderate cerebral palsy

**DOI:** 10.3389/fmed.2024.1295104

**Published:** 2024-06-12

**Authors:** Daniel G. Whitney, Andrea L. Oliverio, Jodi Kreschmer, Shannen Bolde, Edward A. Hurvitz, Ka Kit Wong

**Affiliations:** ^1^Department of Physical Medicine and Rehabilitation, University of Michigan, Ann Arbor, MI, United States; ^2^Institute for Healthcare Policy and Innovation, University of Michigan, Ann Arbor, MI, United States; ^3^Division of Nephrology, Department of Internal Medicine, University of Michigan, Ann Arbor, MI, United States; ^4^Division of Nuclear Medicine, Department of Radiology, University of Michigan, Ann Arbor, MI, United States

**Keywords:** cerebral palsy, adults, glomerular filtration rate, GFR, eGFR, mGFR

## Abstract

**Objective:**

Despite the need, measuring glomerular filtration rate (mGFR) is not routinely performed for adults with cerebral palsy (CP), possibly due to unknown feasibility given the secondary complications of CP. This study aimed to assess the feasibility and reliability of mGFR and explore factors associated with eGFR-mGFR discordance among young adults with mild-to-moderate CP.

**Methods:**

This single-center, cross-sectional study included 18- to 40-year-olds with CP gross motor function classification system (GMFCS) I-III. The participants were excluded if they were pregnant/lactating, had cognitive impairments, or had contraindications to mGFR. A routine clinical protocol for mGFR and eGFR was used. mGFR feasibility was assessed based on the number of participants who completed testing. mGFR reliability was assessed using the coefficient of variation (CV) across the four 30 min intervals. The association between age, sex, and GMFCS and the percentage of eGFR-mGFR discordance was assessed.

**Results:**

Of the 19 participants enrolled, 18 completed the testing [mean age (SD), 29.9 (7.4) years, *n* = 10 female participants, *n* = 10/3/5 for GMFCS I/II/III] and most (*n* = 15) of the participants had an mGFR >90 mL/min; 14 participants (77.8%) had a CV <20%, 2 had a CV between 20 and 25%, and 2 had a CV >50%. eGFR overestimated mGFR by a median (interquartile range) of approximately 17.5% (2–38%); the full range of mis-estimation was −20.5 to 174.3%. Increasing age and GMFCS levels exhibited notable, but weak-to-modest, associations with a larger eGFR-mGFR discordance.

**Discussion:**

Obtaining mGFR was feasible and reasonably reliable within this small sample. eGFR overestimated mGFR by a notable amount, which may be associated with patient-level factors.

## 1 Introduction

Emerging research highlights that chronic kidney disease is a major concern for adults with cerebral palsy (CP) (1–4). However, the current clinical methods, often utilizing creatinine-based estimates of glomerular filtration rate (GFR), can overestimate kidney function at the patient level leading to an underestimation of the true extent of the chronic kidney disease burden in the adult population with CP (1).

Chronic kidney disease is defined by abnormalities of kidney structure or function for 3 months or more, including decreased GFR. The gold standard to assess GFR is by measuring GFR (mGFR) via clearance of an exogenous marker. However, the clinical mGFR test is cumbersome, so GFR is often estimated (eGFR), mostly using the serum biomarker, creatinine, where higher creatinine levels reflect poorer kidney function (5–7). Creatinine is a byproduct of muscle metabolism, and its serum level correlates with muscle mass (8). The low muscle mass in CP results in low levels of creatinine and produces high eGFR values, which is exacerbated for adults with a greater severity of CP (9). This can lead to the erroneous interpretation that kidney function is better than it actually is when using standard eGFR equations (1).

Clinical guidelines recommend mGFR testing when eGFR is suspected to be inaccurate in CP (10). However, in a recent 20-year period at a major medical institute, fewer than 10 adults with CP had an mGFR test performed (1). Measured GFR tests are time-consuming and cumbersome for patients and thus not often utilized despite guideline recommendations; clinically, they are mostly commonly utilized for rigorously testing potential kidney donors for transplantation. Clinical testing of mGFR may also be difficult to perform due to secondary complications of CP, such as bladder complications or issues that limit adequate hydration (e.g., dysphagia) (11–15). A reliable mGFR requires maintaining oral hydration throughout the test and ensuring complete bladder evacuation. Lack of mGFR testing in individuals with CP is anticipated to be widespread and is likely due to a lack of awareness of the need to accurately assess kidney function and unknown feasibility of performing the mGFR test. Thus, understanding the feasibility and reliability of mGFR among adults with CP is critical for future research. This includes efforts to validate accurate, less cumbersome, and clinically useful equations for eGFR designed specifically for people with CP and/or to adapt mGFR protocol such that they are less time-consuming and cumbersome for people with CP.

The primary objective of this study was to determine the feasibility of obtaining mGFR and its reliability among young adults, aged 18–40 years, with mild-to-moderate CP. The secondary objective was to identify reasonable accommodations to enhance the mGFR test. Exploratory analyses examined the association between key patient factors and the extent of eGFR-mGFR discordance. Additionally, these analyses assessed whether decreasing test time yielded representative estimates of mGFR for research and clinical considerations.

## 2 Methods

### 2.1 Study design and inclusion/exclusion criteria

This study was supported by an R03 grant from the Eunice Kennedy Shriver National Institute of Child Health and Human Development (R03HD105589). The study was designed to assess the feasibility of obtaining mGFR among young adults aged 18–40 years with mild-to-moderate CP as the primary outcome. All other outcomes were considered secondary, tertiary, and exploratory (hypothesis-generating). A formal sample size calculation can be inappropriate due to the preliminary nature of feasibility studies (16). It has been recommended to recruit at least 12 participants for feasibility studies without formal calculations (17). To optimize the sample size in relation to the available resources, this study was designed to include up to 20 participants.

This was a cross-sectional study approved by the University of Michigan Institutional Review Board, and written informed consent was obtained from each participant. Recruitment and data collection took place from January 2022 to June 2023. Adults aged 18–40 years with any type of CP with gross motor function classification system (GMFCS) of I–III (mild to moderate) (18) were included. In this early research phase, we opted for the age range of 18–40 years as this might be an important window for early prevention. We opted for mild-to-moderate CP to limit complexity to begin understanding the mGFR feasibility.

The exclusion criteria included pregnancy, lactation, urinary tract infections symptoms, or contraindications to the mGFR test including oral-motor issues (e.g., dysphagia) or use of a urinary catheter. Individuals with cognitive impairment that would limit the ability of the individual to understand the study, follow directions, or properly consent to study participation were also excluded.

### 2.2 Setting and radiation exposure

This study used the University of Michigan Nuclear Medicine & Molecular Imaging protocol, which uses a slightly modified version (5) of an established clinical protocol (19–22). The effective radiation dose [presented as millirem (mrem) and milliSieverts (mSv)] for the study protocol is 90 mrem (0.90 mSv), which is less than the annual 300 mrem (3.0 mSv) of naturally occurring “background” radiation.

### 2.3 Recruitment methods

This study used various types of convenience sampling for recruitment, which included obtaining participants from clinics focused on the care of adults with CP, from other ongoing studies, and through referrals from study participants and research staff. Additionally, study flyers were distributed to potentially eligible individuals to introduce the study and provide contact information for the research team if they were interested. This study also used non-convenience sampling. The study flyers were emailed to 18- to 40-year-olds diagnosed with CP and having a clinical encounter between August 2021 and August 2022, identified from the Medical Center’s electronic medical records. The study information was also posted on UM Health Research,^1^ wherein individuals both within and outside the University of Michigan can search for studies posted and contact research teams if interested.

### 2.4 ^125^I-Iothalamate to assess mGFR

Timed clearance of exogenous markers of known quantities by the kidneys is used to assess mGFR. A radioactively labeled material, ^125^I-Iothalamate, is injected subcutaneously to allow a sustained equilibrium of radiotracer concentration in the vascular space over the duration of the study. ^125^I-Iothalamate clearance by the kidneys demonstrates minimal bias, good accuracy, and good test–retest reliability [coefficient of variation (CV) = 6.3–8.5%] (5, 23–25), as well as high correlation (90–95%) with other exogenous markers (e.g., inulin and iohexol) for mGFR (26, 27), and has been widely adopted as the clinical gold standard for mGFR assessment (28).

Following an overnight fast and prior to injections or intravenous line placement, a urine sample was collected as the “background.” An intravenous line was placed, and a blood sample was taken as the “background.” A quantity of 35 microcuries of ^125^I-Iothalamate was injected subcutaneously. After 60 min, the blood and urine samples were collected as “time 0,” which is followed by four timed collections of urine and serum every 30 min (time 1–4).

### 2.5 mGFR analysis

Blood samples were centrifuged at 3,200 rpm for 8 min. Duplicate 0.5 mL samples of blood and urine samples were pipetted to assess the counts of the exogenous marker per minute in each sample using the Wizard^2^ Autowell Detector Gamma Counter (PerkinElmer, Waltham, Massachusetts, United States). The urine and serum counts for time 0–4 were corrected by subtracting the “background” counts to normalize to each participant. The mGFR per time interval and the average across time 1–4 was calculated using a standard formula, which includes the DuBois formula (29) for body surface area (0.007184 × [height (m)^0.725^] × [body mass (kg)^0.425^]). The coefficient of variation (CV) of the four mGFRs is used to assess the reliability of the test. The interpretation of CV% is subjective and may vary depending on the clinical goals. The clinic considers a CV of ≤20% to indicate a reliable mGFR test for kidney transplant donors.

### 2.6 ^99m^Tc-DTPA to assess split kidney function

Immediately following the mGFR test, participants underwent kidney imaging using a gamma camera renogram to assess split kidney function via filtering the exogenous marker, technetium-99 m diethylenetriaminepenta-acetic acid (^99m^Tc-DTPA), injected through the IV line. The outcome measure is the percentage of kidney function attributed to the left and right kidneys, where values of 45–55% were considered a typical range.

### 2.7 Exit interviews

Within 1 week of the research visit, a semi-structured interview was conducted via telecommunication by a research team member with expertise in conducting qualitative interviews with adults with disabilities. The purpose of this interview was to learn the participants’ perspective about what they liked, did not like, and would change regarding the mGFR protocol. The goal was to inform “reasonable accommodations” that may enhance recruitment and retention for future studies involving mGFR testing and completion of the mGFR test with reliable values. The exit interview was designed to be simple, short (<8 min), and sequester key information needed to inform future study designs.

There were two main questions. The first question was, “Can you talk about any part or parts of the test that you found to be uncomfortable or challenging?” Follow-up questions were pursued based on the participant’s response. The second question was, “In what ways do you recommend we modify the testing process so that it is easier for you or others with cerebral palsy to do?” This process was informal, and key takeaways were documented by the research team as opposed to transcription and formal analysis of qualitative information.

### 2.8 Other characteristics

Age, self-reported sex, gender, race, and ethnicity were collected. Sex and gender were the same for all participants, and thus, only sex is reported. The type of CP, its topographical distribution, and GMFCS were assessed from the medical records where available, through self-report, and/or by the study team with knowledge in describing the CP phenotype. Body mass was measured using a standard clinical weight scale with an assistive frame for holding for balance. Height was obtained from the most recent measurement in the medical record.

The serum from the “background” blood sample was analyzed using standard clinical assays for Comprehensive Metabolic Panel, which includes creatinine and cystatin C analytes. The equation used to calculate eGFR depended on age. For adults aged 18 to 25 years old, the CKiD Under 25 (U25) estimating equations were used, which utilize age, sex, height, and creatinine with and without cystatin C (30, 31). For adults aged 26 and older, the 2021 race-free version of the CKD Epidemiology Collaboration (CKD-EPI) estimating equations was used, which utilizes age, gender, body surface area, and creatinine with and without cystatin C (32). Both sets of estimating equations have a (1) creatinine only (Cr) and (2) creatinine + cystatin C (Cr + CystC) equations, all of which were included in the analysis. For consistency with the mGFR calculation, CKD-EPI eGFR was adjusted to the individual’s body surface area, as previously described (33), as opposed to the standard body surface area of 1.73m^2^ proposed in 1928 (34) that may be less relevant to a modern population and adults with CP (35, 36).

### 2.9 Statistical analysis

Descriptive statistics were used to summarize recruitment methods, feasibility measures, and participant characteristics. Of those that initiated data collection, the feasibility (or “completion rate”) of performing the mGFR test was described as the number of individuals who completed the testing, while the reliability of mGFR was assessed by the CV%. Skewness and kurtosis assessed the normality of distribution for the continuous outcome variables. Mean [standard deviation (SD)] or median [interquartile range (IQR)] was presented based on whether the data were normally or non-normally distributed, respectively. Qualitative data from the exit interviews were summarized and key findings were reported, as were adverse events.

### 2.10 Sensitivity analysis

Measuring height for adults with CP is challenging and not performed at the study visit. Height was therefore taken from the medical records, which may be inaccurate. Body surface area is part of the equation to calculate mGFR. The value of body surface area is more sensitive to errors in height than body mass (see formula above). We therefore examined mGFR after assuming an error of height by ±5% to assess for the possibility of changes in interpretations. We selected ±5% as this degree of error in measurement is anticipated to encompass the vast majority of real-world measurement error.

### 2.11 Exploratory analysis

The discordance between eGFR with the average mGFR was assessed per person as an absolute and relative (%) difference. To provide hypothesis-generating evidence, the association between discordance and age was assessed using simple linear regression via the coefficient of determination (*r*^2^). The association between discordance and sex and GMFCS (I vs. II/III) was assessed using Cohen’s *d* (37). Significance tests and related statistics (e.g., *p*-value) are not reported to avoid misinterpreting exploratory analyses with inference-based interpretations.

For the duration of the ~4- to 5-h mGFR test, the individual is required to remain fasted, which poses a threat to research recruitment, feasibility, and reliability when using this procedure. We examined whether mGFR estimated from fewer time intervals provides a representative estimate of mGFR using all four time intervals. Pearson’s correlation (*r*) from simple linear regression assessed the relationship between mGFR from all 4 time intervals (reference) and the average mGFR from (1) time 1 through time 3, (2) time 1 + time 2, (3) time 1 + time 3, and (4) time 2 + time 3.

Analyses were performed using SAS version 9.4 (SAS Institute, Cary, NC, United States), and a *p-*value of ≤0.05 (two-tailed) was used to determine statistical significance where relevant.

## 3 Results

### 3.1 Recruitment

A total of 752 individuals were contacted regarding the study (Supplementary Table S1), of which, 44 were screened; the remaining708 did not respond or expressed interest but were unable to connect with the study team for screening. Of those screened, *n* = 17 were not eligible (*n* = 10) or were unable to consent due to cognitive impairment (*n* = 7). Of those eligible, *n* = 8 did not consent or later withdrew after consenting due to busy schedules or they stopped responding to the study team.

### 3.2 Feasibility

A total of 19 participants enrolled and initiated the data collection. One individual opted out of the ^99m^Tc-DTPA scan but completed all other testing and was considered to have completed the study. One individual did not complete the mGFR or ^99m^Tc-DTPA due an adverse event described below. The completion rate of the mGFR test (*n* = 18) for those who started data collection (*n* = 19) was 94.7%.

Descriptive characteristics for the sample that completed the study are presented in Table 1. Fasted serum values from the routine clinical assays are shown in Supplementary Table S2. All participants had creatinine and cystatin C values within the normative range. All participants had split kidney function values within the normal range of 45–55%, except one participant (30-year-old white woman, GMFCS I, mGFR = 72.6, 22.0% CV) where the right and left kidneys contributed to 59.0 and 41.0% of total kidney function, respectively (data not shown).

**Table 1:** Descriptive characteristics of the cohort that completed the study (*n* = 18).

Age, mean (SD)	29.9 (7.4)
**Sex, % (*n*)**	
Male	44.4 (8)
Female	55.6 (10)
**Race, % (*n*)**	
Black	11.1 (2)
White	88.9 (16)
**GMFCS, % (*n*)**	
I	55.6 (10)
II	16.7 (3)
III	27.8 (5)
**Type of cerebral palsy, % (*n*)**	
Spastic hemiplegia	22.2 (4)
Spastic diplegia	72.2 (13)
Ataxia	5.6 (1)
Body mass (kg), mean (SD)	72.9 (18.9)
Height (m), mean (SD)	1.67 (0.11)
Body mass index (kg/m^2^), mean (SD)	26.1 (5.3)

SD, standard deviation; GMFCS, gross motor function classification system.

Of the 18 individuals who completed the mGFR test, 83.3% (*n* = 14) had a CV ≤20%. Meanwhile, two individuals had a CV of 22.0 and 23.0%, and another two had a CV >50%. Four participants with CV >20% were all white and GMFCS I but were heterogeneous with regard to age (20.2 to 35.4 years old), sex (*n* = 1 male participant and *n* = 3 female participants), BMI (23.9 to 33.7 kg/m^2^), type of CP (*n* = 3 spasticity and *n* = 1 ataxia), and start time of testing (*n* = 3 07:30 and *n* = 1 10:30). All four participants met the fasting and pre-testing criteria (e.g., caffeine and NSAID restriction) and achieved the “desirable” minimal urinary flow rate of ≥4 mL/min for each time interval. The male participant experienced difficulty urinating at the beginning of the test (akin to paruresis symptoms) but maintained water consumption. The urinary flow rate by time 4 (17.0 mL/min) became exceedingly faster than the prior time points (7.0 to 11.8 mL/min), which led to a very high mGFR value by time 4 (215.6 units) compared to the prior time points (such as 76.7, 64.6, and 128.4 units). However, the CV >20% was not explained by variations in the urinary flow rate throughout the test for the three female participants, and no other testing parameters were noted that may help to explain the higher CV values observed in other three participants.

The results of the testing, including the CV%, eGFR, and the discordance between eGFR and the average mGFR, are presented in Table 2 for the full sample that completed the study (*n* = 18) and the subset with a CV ≤20% (*n* = 14).

**Table 2:** Results of the glomerular filtration rate tests and discordance for the full cohort that completed the study and those with a coefficient of variation (CV) ≤20%.

	All (*n* = 18)	Those with CV ≤20% (*n* = 14)
	Median (IQR)	Median (IQR)
**mGFR and eGFR results**		
*mGFR*		
Time 1	107.2 (89.6, 114.1)	109.2 (99.0, 114.1)
Time 2	100.0 (88.8, 107.2)	102.9 (95.1, 107.2)
Time 3	96.7 (89.5, 104.5)	97.4 (91.0, 104.5)
Time 4	93.5 (84.9, 102.5)	93.5 (87.5, 102.5)
Average of time 1–4	100.2 (95.5, 106.6)	100.2 (96.2, 103.9)
CV of time 1–4 (%)	9.5 (5.1, 15.6)	8.2 (4.9, 10.5)
eGFR, creatinine only equation	118.0 (101.3, 126.6)	118.0 (104.4, 134.9)
eGFR, creatinine + cystatin C equation	112.8 (101.9, 132.6)	112.8 (104.2, 135.8)
**mGFR and eGFR discordance**		
*Absolute difference, eGFR—mGFR (GFR units)*		
eGFR, creatinine only equation	17.1 (2.5, 39.6)	17.1 (10.1, 35.0)
eGFR, creatinine + cystatin C equation	15.6 (2.5, 41.2)	15.6 (3.2, 37.3)
*Percent difference, eGFR—mGFR (%)*		
eGFR, creatinine only equation	17.6 (2.4, 37.0)	17.6 (9.4, 34.0)
eGFR, creatinine + cystatin C equation	17.4 (2.3, 38.1)	17.4 (3.1, 36.6)

mGFR, measured glomerular filtration rate; eGFR, estimated glomerular filtrate rate; IQR, interquartile range.

### 3.3 Exit interviews

Most participants mentioned the test was easy and doable. Specific barriers noted often related to the clinical space. Accommodations for individuals with CP were recommended. For example, the restroom was approximately 15 meters down the hall. Several participants suggested that a room with its own bathroom would be more comfortable and easier to navigate. The clinic uses bottled water for measurement purposes. It was noted that a straw for the water bottles would help those with motor challenges for coordinating drinking from a water bottle. A few participants noted that the length of the test was tiring, especially as they could not eat for the duration of the test and would prefer if the test could be shortened.

### 3.4 Sensitivity analysis

When height was set to 5% lower and higher than the recorded height, the percent change in mGFR increased on average (SD) by 3.80% (0.05) and decreased on average (SD) by 3.47% (0.04), respectively, with minimal variation between participants (*n* = 18) (Supplementary Figure S1).

### 3.5 Adverse events

One participant (33-year-old woman, GMFCS III) had a vasovagal event after the IV insertion. However, she fully recovered after lying supine for 15 min and opted to continue the test. There were no further issues during the remainder of the testing or data collection. One participant (24-year-old woman, GMFCS I) felt nauseous approximately 30 min after the IV insertion and fell when reaching for a trashcan. She was admitted to the Emergency Room near the clinic where they labeled the event as “unspecified syncope” after performing a thorough evaluation.

### 3.6 Exploratory analysis: factors associated with the discordance between eGFR and mGFR

There was evidence of an outlier for percent discordance from one participant (>3 standard deviations above group mean): female participant, mid-30s, GMFCS I, average mGFR = 45.2, mGFR CV = 50.3%, eGFR of 124.0 (Cr equation) and 131.8 (Cr + CystC equation). Given the small sample size, the following analyses were performed with the full sample (*n* = 18) and after omitting the outlier (*n* = 17). There was a weak positive association between age and percent discordance for both equations (Cr and Cr + CystC, *r*^2^ = 0.15 and 0.18, respectively) (Supplementary Figure S2). The association became stronger with a moderate effect estimate after removing the outlier and was similar between Cr and Cr + CystC equations (*r*^2^ = 0.29).

After removing the one outlier (female participant, GMFCS I), the percent discordance was similar between male and female participants and higher for GMFCS II/III vs. I (Supplementary Table S3).

### 3.7 Exploratory analysis: shorter intervals to estimate average mGFR

**Figure 1:**
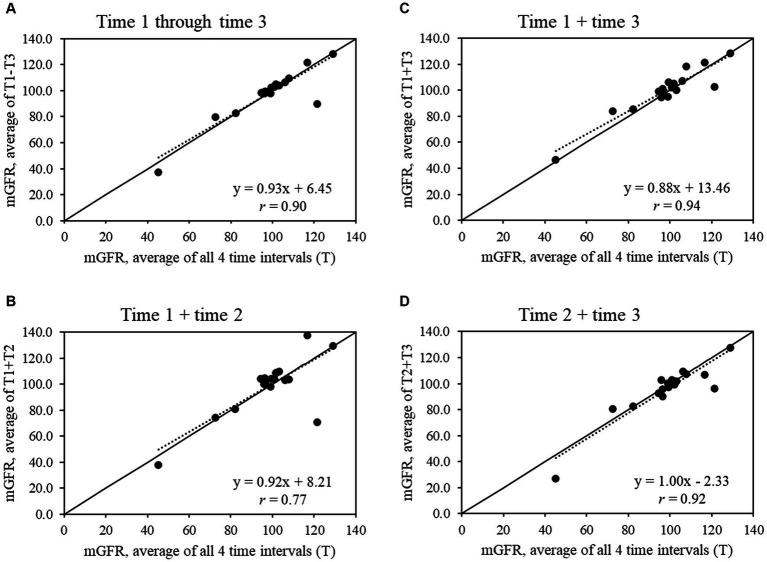
Scatter plots show the relationships between average measured glomerular filtration rate (mGFR) using all 4 time intervals and average mGFR using time intervals **(A)** 1 through 3, **(B)** 1 and 2, **(C)** 1 and 3, and **(D)** 2 and 3 for the full sample that completed mGFR testing (*n* = 18).

**Figure 2:**
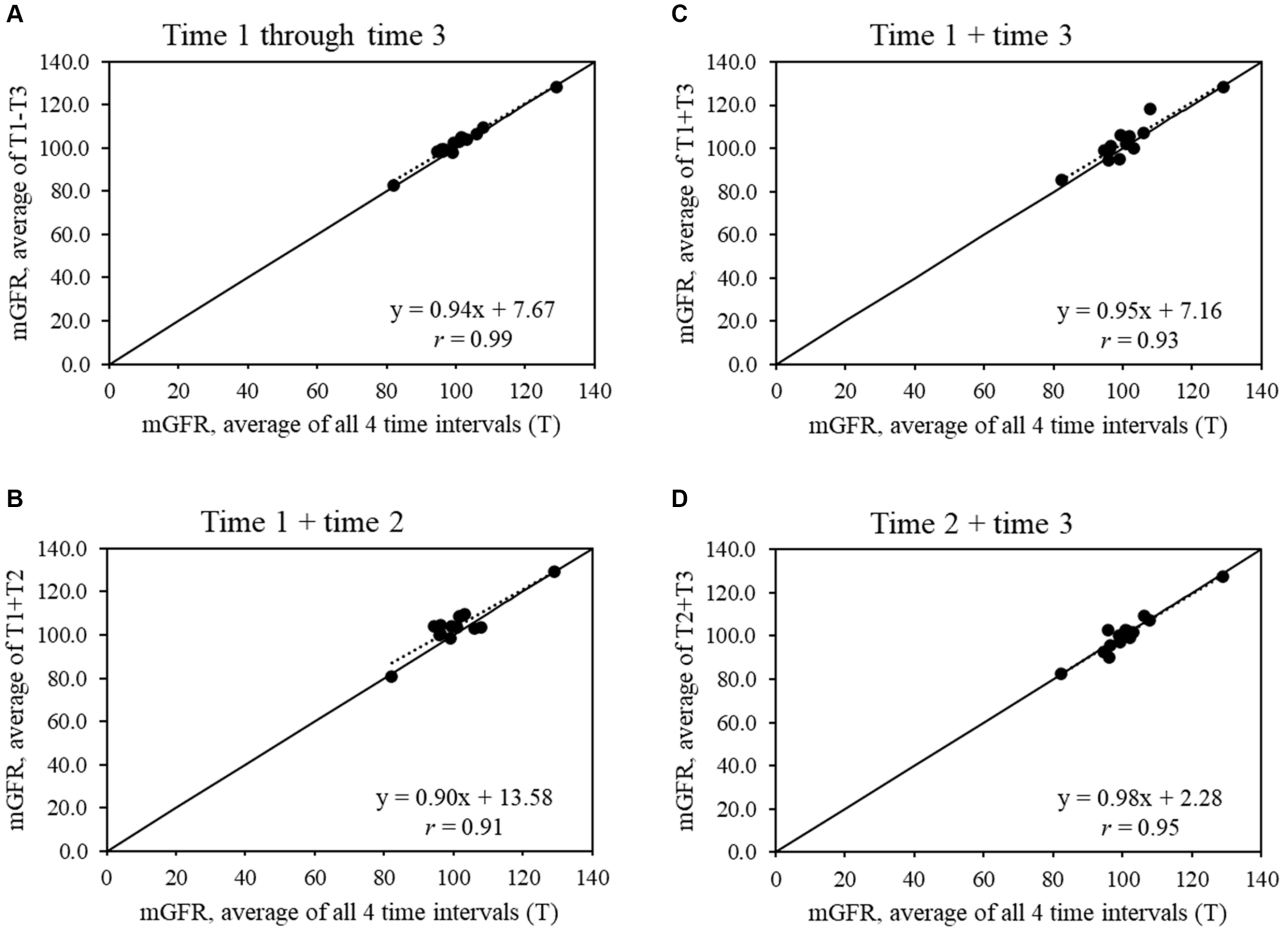
Scatter plots show the relationships between average measured glomerular filtration rate (mGFR) using all 4 time intervals and average mGFR using time intervals **(A)** 1 through 3, **(B)** 1 and 2, **(C)** 1 and 3, and **(D)** 2 and 3 for the sample that had an mGFR CV ≤20% (*n* = 14).

For the full sample (Figure 1), all shorter time intervals showed strong to very strong correlations with the average mGFR from all 4 time intervals (*r* = 0.77 to 0.94), while the strength of the correlations increased in the subset of 14 participants with a CV ≤20% (*r* = 0.91 to 0.99) (Figure 2).

## 4 Discussion

The findings from this small sample of young adults with mild-to-moderate CP suggest that performing a routine clinical mGFR test was feasible for this group. The risk of adverse events in this study was found to be 10.5% (equating to 2 out of the 19 participants who initiated the test), which has been subjectively assessed as “not largely different” than non-CP patients by the clinical team that routinely performs this test. The adverse events involved vasovagal and nausea symptoms following the initial IV insertion, which are common adverse events for this test.

The clinic uses a threshold of CV ≤20% to indicate a reliable mGFR for kidney transplant donor patients. Based on CV ≤20%, 14 out of the 18 participants, or 77.8%, met this criterion. For clinical scenarios that may not require such a stringent cutoff, the participants with a CV of 22.0 and 23.0% may be considered to have a reasonably reliable test, at least for general clinical assessment and health management. Depending on the clinical goals, this suggests that approximately 78–89% of this sample had a reliable mGFR.

The generalizability of the findings is not known. The clinical environment was not designed or modified for patients with physical disabilities and is considered standard. The technician that performed the tests lacked formal training or experience in caring for people with physical disabilities. These factors generalize to many clinics that perform the mGFR test, suggesting that the feasibility and reliability findings may translate to other standard clinical environments, at least to some extent. It is important to note that the feasibility and reliability findings from this study may not translate to middle-aged and older adults with CP or adults with more severe forms of CP at any age. With greater severity of CP comes more medical and logistical complexity, which can complicate the feasibility and decrease the reliability of the mGFR test.

The majority of this study’s sample found the mGFR test to be relatively easy, but a shorter length of testing time was a main feedback item. For research, the ~4- to 5-h mGFR test can pose challenges in recruitment and retention, especially for longitudinal studies with repeat mGFR tests. In our exploratory analysis, we found that time interval 1 +3 or 1 –3 had the strongest correlation with slopes close to 1.00 in the full sample and the subset with CV ≤20%. These findings provide preliminary evidence that, if needed, the mGFR test may be able to be shortened to 3 time intervals to balance rigor in data collection against participant recruitment, engagement, and retention. Research with larger sample sizes will be needed to confirm whether the mGFR test can be shortened without losing information on kidney function.

The creatinine-based eGFR equation is known to overestimate mGFR for populations with low muscle mass. The use of cystatin C shows promise as it is less-dependent upon muscle mass (38). Among individuals with spinal cord injury, cystatin C and its eGFR equations better capture kidney function than creatinine and its eGFR equations (39). However, cystatin C is expressed by adipose tissue, with expression levels increasing with enlarged adipocytes (40). Individuals with vs. CP have higher total body fat relative to their shorter stature and greater adipose tissue and fatty infiltration within the visceral and musculoskeletal depots, compared to individuals without CP (41–44). In this study, eGFR overestimated mGFR for the majority of participants similarly regardless of whether the equation used was creatinine only or creatinine + cystatin C, which may deviate from other groups where cystatin C is the better biomarker as opposed to creatinine. The exploratory analysis suggests a weak-to-modest effect of older age and higher GMFCS levels on greater eGFR-mGFR discordance, but further research is required to confirm these associations. The outlier for the percent discordance analysis could be attributed to the participant having a disproportionately altered body composition (e.g., low muscle mass and high fat mass) resulting in a higher eGFR. Alternatively, it could be due to difficulties with the hydration and voiding protocol, resulting in a lower mGFR.

It is important to note that most participants in this study had an mGFR value >90 and were within normative limits for their age and sex, which indicates normal or high kidney function (45). Furthermore, the sensitivity analysis that varied height found no significant difference in mGFR that would translate to meaningful clinical misinterpretations of mGFR. Two individuals had an mGFR between 60 and 89 (mGFR = 72.6 and 82.2), indicating chronic kidney disease stage 2, while one individual had an mGFR of 45.2, indicating chronic kidney disease stage 3 if these findings were persistent over 3 months or more. Importantly, the eGFR results from the creatinine only equation (creatinine + cystatin C equation) for these individuals were within normative limits (i.e., >90) and overestimated the mGFR by 20.6–174.3% (15.7–191.6%). In the clinical setting, this would misclassify the stage of chronic kidney disease, leading to missed opportunities for prevention and possibly treatment. Unfortunately, eGFR serves as a main indicator for a nephrology referral by primary care physicians or other specialists. Since kidney dysfunction does not typically result in pain or other noticeable symptoms, nephrology referrals for additional assessments of possible CKD and early prevention or treatment of kidney dysfunction may be missed for many adults with CP.

This study has limitations in addition to those mentioned above. The generalizability of findings is unknown. The sample size was small. The recruitment methods casted a wide net, but only a small fraction of individuals were screened and enrolled. The authors are aware of different mGFR protocols, such as those utilizing ^99m^Tc-DTPA and 2 time-point blood sampling (Russell method). However, we chose the protocol used in this study as it is clinically regarded as more accurate and valid. This study did not measure aspects of nutritional status, which could impact the results. However, significant malnutrition is less common for the GMFCS levels included in this study.

In conclusion, clinical mGFR testing was feasible and reliable among this small sample of young adults with mild-to-moderate CP. Consistent with prior study, eGFR using creatinine only equations overestimated mGFR in this sample. However, eGFR using creatinine + cystatin C equations also overestimated mGFR to a similar extent in this sample, which may differ from other clinical groups. Future research is needed to assess the feasibility of mGFR among older and/or more severe patients with CP and to develop CP-specific eGFR equations that better capture mGFR.

## Data availability statement

The raw data supporting the conclusions of this article will be made available by the authors, without undue reservation.

## Ethics statement

The studies involving humans were approved by the University of Michigan Institutional Review Board. The studies were conducted in accordance with the local legislation and institutional requirements. The participants provided their written informed consent to participate in this study.

## Author contributions

DW: Writing – original draft, Visualization, Validation, Supervision, Project administration, Methodology, Investigation, Funding acquisition, Formal analysis, Conceptualization. AO: Writing – review & editing, Methodology, Investigation, Funding acquisition, Conceptualization. JK: Writing – review & editing, Project administration, Data curation. SB: Writing – review & editing, Project administration, Data curation. EH: Writing – review & editing, Methodology, Investigation, Funding acquisition, Conceptualization. KW: Writing – review & editing, Supervision, Methodology, Investigation, Funding acquisition, Conceptualization.

## Footnotes

^1^https://umhealthresearch.org/
