# Supplementary material for: Feasibility and reliability of measured glomerular filtration rate with [I125]-iothalamate among young adults with mild-to-moderate cerebral palsy
Source: Front Med (Lausanne). 2024 Jun 12;11:1295104. doi: 10.3389/fmed.2024.1295104 (PMC11199527; doi:10.3389/fmed.2024.1295104)
Supplement: Supplementary file 1 [file Data_Sheet_1.docx]

Supplementary Material

**Supplementary Table 1.** Recruitment methods.

| Recruitment method | Date of method implementation | # contacted | # screened | # eligible | # enrolled | # started study | # completed study |
| --- | --- | --- | --- | --- | --- | --- | --- |
| Mailchimp email newsletter to patients at Michigan Medicine | Email sent on the following dates: 08/10/2022, 08/17/2022, 08/30/2022, 09/09/2022, 09/16/2022, 09/26/2022 | ~720* | 9 | ** | 3 | 3 | 3 |
| University of Michigan Health Research website | Research website published 08/17/2022 | 4 | 1 | ** | 0 | 0 | 0 |
| Friend of research staff or friend referral | Started 05/2022 | 5 | 4 | ** | 2 | 2 | 2 |
| Referral from CP clinics or other studies by research team | Started 05/2022 | 52 | 22 | ** | 10 | 9 | 8 |
| Email blast with informational flyer | Email sent on the following dates: 11/08/2022, 01/12/2023 | 330* | 8 | ** | 8 | 5 | 5 |
|  | Total | n=752* | n=44 | n=27 | n=23 | n=19 | n=18 |

*There was an overlap among individuals contacted via the Mailchimp and Email blast recruitment methods. The reported sample size is the number of unique individuals.

**The level of detail was not documented, and the total sample is presented instead.

**Supplementary Table 2.** Fasted serum values from routine clinical assays, Comprehensive Metabolic Panel and Cystatin C, for the cohort that completed the study (n=18).

|  | Mean (SD) | Full range | Notes |
| --- | --- | --- | --- |
| Sodium | 139.1 (2.3) | 134 - 143 | Low (n=2) |
| Potassium | 4.0 (0.4) | 3.5 - 4.7 |  |
| Chloride | 104.7 (2.6) | 99 - 109 | Elevated (n=1) |
| Carbon dioxide | 26.8 (2.4) | 21 - 31 |  |
| Urea nitrogen | 11.6 (2.6) | 7 - 16 |  |
| Creatinine | 0.7 (0.1) | 0.51 - 0.95 |  |
| Glucose | 86.3 (8.5) | 72 - 101 |  |
| Calcium | 9.3 (0.3) | 8.5 - 9.7 | Low (n=1) |
| Protein | 7.0 (0.3) | 6.3 - 7.4 |  |
| Albumin | 4.4 (0.3) | 3.9 - 4.8 |  |
| AST | 23.4 (11.4) | 12 - 52 | Elevated (n=2) |
| ALT | 25.8 (22.4) | 9 - 99 | Low (n=2); elevated (n=1) |
| Alkaline phosphatase | 67.9 (21.8) | 43 - 133 | Elevated (n=1) |
| Total bilirubin | 0.7 (0.4) | 0.3 - 1.9 | Elevated (n=1) |
| Anion gap | 7.5 (2.0) | 4 - 10 |  |
| Cystatin c | 0.8 (0.1) | 0.58 - 0.90 |  |

**Supplementary Table 3.** Exploratory analysis. Group differences in the discordance between measured and estimated glomerular filtration rate by sex and gross motor function classification system (GMFCS) before and after removing one outlier.

|  | Creatinine only equation | | Creatinine + cystatin c equation | |
| --- | --- | --- | --- | --- |
|  | Mean (SD) | *d* | Mean (SD) | *d* |
| n=18 |  |  |  |  |
| Sex |  |  |  |  |
| Male (n=8) | 17.5 (20.4) | Reference | 18.0 (23.4) | Reference |
| Female (n=10) | 33.6 (55.2) | 0.37 | 34.1 (60.8) | 0.33 |
| GMFCS |  |  |  |  |
| I (n=10) | 30.2 (57.6) | Reference | 31.3 (63.1) | Reference |
| I/II (n=8) | 21.8 (13.5) | -0.19 | 21.4 (17.1) | -0.20 |
| n=17 |  |  |  |  |
| Sex |  |  |  |  |
| Male (n=8) | 17.5 (20.4) | Reference | 18.0 (23.4) | Reference |
| Female (n=9) | 18.0 (26.1) | 0.02 | 16.6 (26.8) | -0.05 |
| GMFCS |  |  |  |  |
| I (n=9) | 14.2 (29.2) | Reference | 13.5 (30.5) | Reference |
| I/II (n=8) | 21.8 (13.5) | 0.32 | 21.4 (17.1) | 0.31 |

*d*, Cohen’s *d*, where 0.20, 0.50, and 0.80 represent small, medium, and large effect sizes respectively.


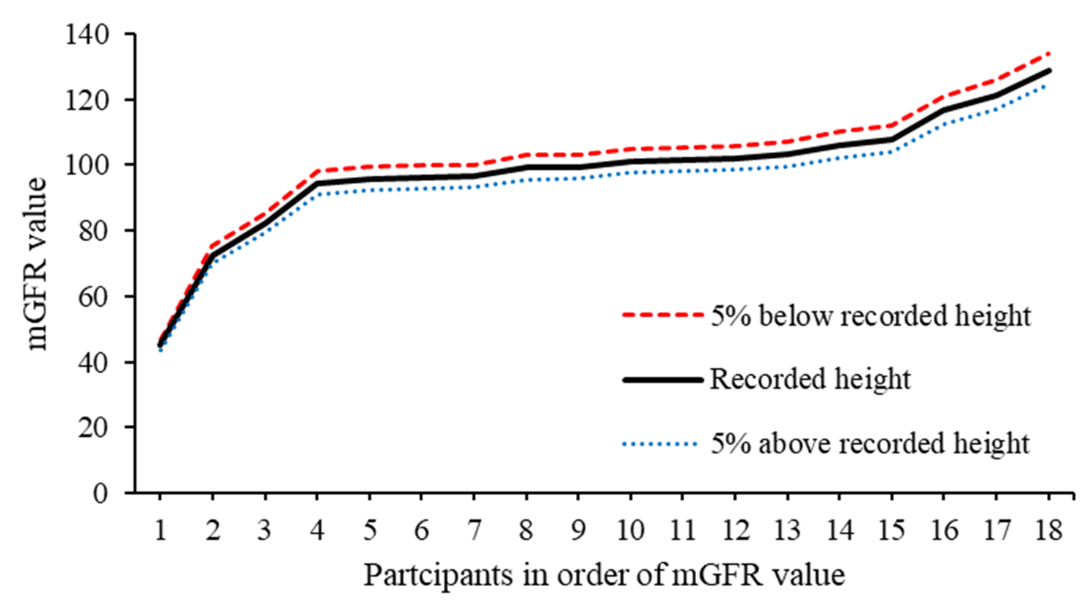


**Supplementary Figure 1.** Average measured glomerular filtration rate (mGFR) using the height recorded in the medical records and a sensitivity analysis after varying height by ±5% to assess for the possibility of changes in interpretations of mGFR.


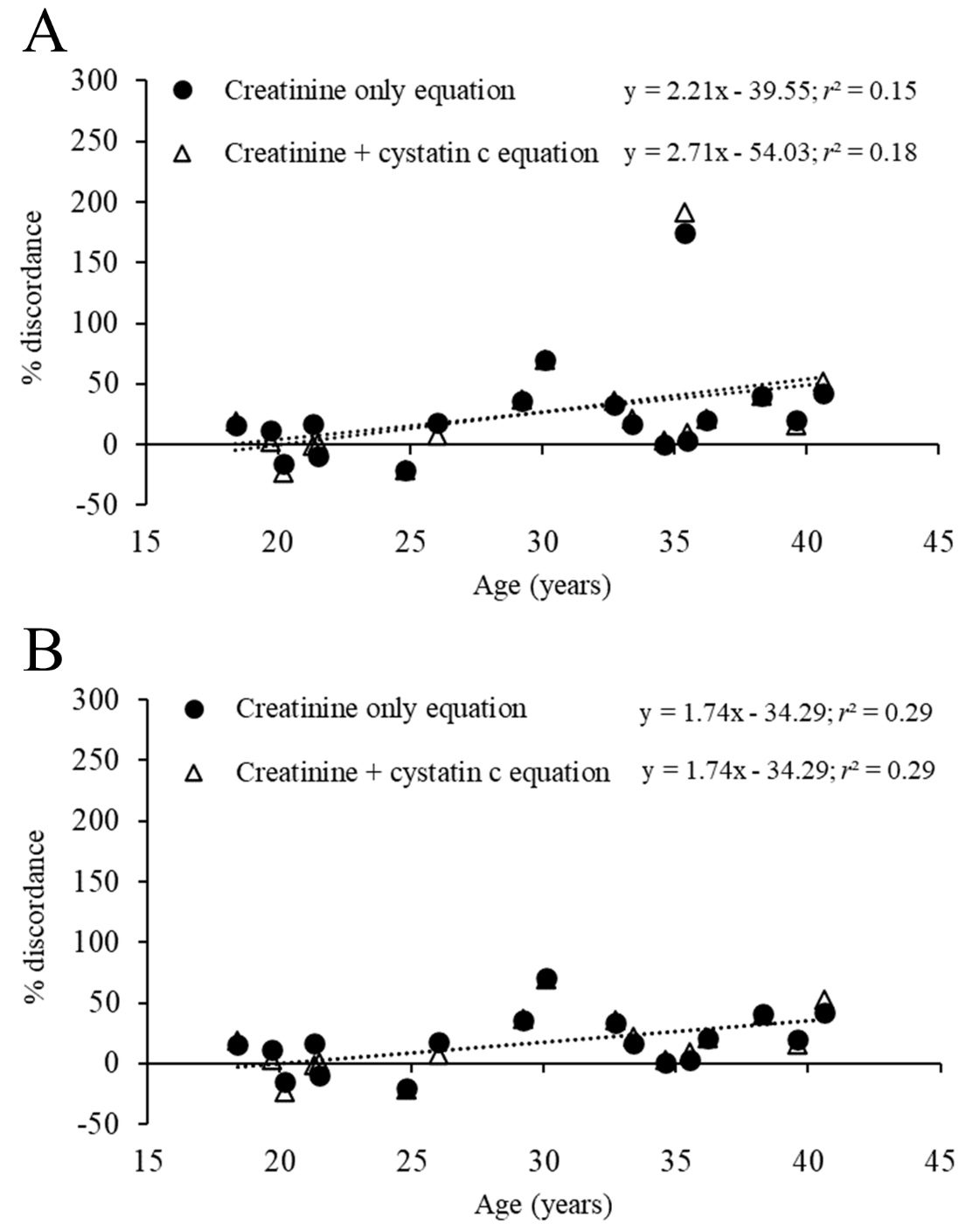


**Supplementary Figure 2.** Scatter plots show the association between age and percent discordance of estimated glomerular filtration rate (eGFR) and measured GFR (mGFR) before (A) and after (B) removing the one outlier. eGFR was assessed using the creatinine only equation and creatinine + cystatin c equation.
